# Quantitative Assessment of Upper Limb Movement in Post-Stroke Adults for Identification of Sensitive Measures in Reaching and Lifting Activities

**DOI:** 10.3390/jcm12093333

**Published:** 2023-05-08

**Authors:** Monika Błaszczyszyn, Agnieszka Szczęsna, Mariusz Konieczny, Paweł Pakosz, Stefan Balko, Zbigniew Borysiuk

**Affiliations:** 1 Department of Physical Education and Sport, Faculty of Physical Education and Physiotherapy, Opole University of Technology, Prószkowska 76, 45-758 Opole, Poland; 2 Department of Computer Graphics, Vision and Digital Systems, Faculty of Automatic Control, Electronics and Computer Science, Silesian University of Technology, Akademicka 16, 44-100 Gliwice, Poland; 3 Department of Physical Education and Sport, Faculty of Education, J.E. Purkyne University, 400 96 Usti nad Labem, Czech Republic

**Keywords:** upper limb, quantification of movement, post-stroke movement features

## Abstract

Background: The assumption of this work is the achievement of objective results of the movement structure, which forms the basis for in-depth analysis and, consequently, for determining the upper limb movements that are most affected by stroke compared to healthy people. Methods: An analysis of relevant and systematically identified features of upper limb movement in post-stroke adults is presented based on scalable hypothesis tests. The basic features were calculated using movements defined by the x, y, and z coordinates (i.e., 3D trajectory time series) and compared to the results of post-stroke patients with healthy controls of similar age. Results: After automatic feature selection, out of the 1004 common features of upper limb movement, the most differentiated were the upper arm movements in reaching kinematics. In terms of movement type, movements in the frontal plane (shoulder abduction and adduction) were the most sensitive to changes. The largest number of discriminating features was determined on the basis of acceleration time series. Conclusions: In the 3D assessment of functional activities of the upper limb, the upper arm turned out to be the most differentiated body segment, especially during abduction and adduction movements. The results indicate a special need to pay attention to abduction and adduction movements to improve the activities of daily living of the upper limbs after a stroke.

## 1. Introduction

Stroke is the leading cause of acquired disability in adults worldwide [1]. A total of 2 million patients are diagnosed with stroke each year in China, over 1 million in Europe, and over 690,000 in the United States. It is estimated that these numbers will increase dramatically due to the aging of societies [2,3].

Stroke survivors often exhibit upper limb motor impairments that affect the performance of their functional activities such as reaching [4]. In clinical settings, motor impairment is mainly assessed using tools based on the examiner’s observations, such as the Fugl-Meyer Assessment and Action Research Arm Test for the upper limbs. Both are important instruments for clinical evaluation; however, their results are strongly influenced by the observer’s experience [5,6]. Moreover, these tools mainly focus on task performance without analyzing the way a given task is performed; therefore, they fail to describe the specific types of motor deficits [7,8].

The kinematic analysis enables an accurate and objective evaluation of motor functions by providing objective and quantitative movement structure information [9,10]. However, it requires special equipment and more complex identification and interpretation of kinematic indicators. Optoelectronic systems based on visual markers are often considered the gold standard for kinematic analysis due to their high accuracy and reliability [11,12]. Kinematic assessments are expected to allow the precise quantification of motion and distinguish between restitution and compensation [13,14,15].

Current studies underpin the necessity of assessing different relevant functional movements close to real-world conditions rather than relying solely on clinical measures [16]. Motor control research has shown that upper-arm reach movements tend to maintain a uniform pattern based on a straight hand path with a smooth bell-shaped velocity profile. Therefore, in order to generate such a tracking reference, the minimal jerk trajectory method described in [17,18] was implemented. The tracking reference has been successfully used in rehabilitation assisting devices [19,20].

Despite its many advantages, the standard clinical measures that are commonly used by therapists do not account for movement quality and are, therefore, insensitive to detection or changes due to restitution or behavioral compensation. Technologies enabling the objective measurement of motion kinematics and kinetics have been suggested as the best way to solve this problem [21]. Considering the above data, there is a need to implement more innovative and advanced methods of diagnosing, monitoring, and rehabilitation of post-stroke patients. To a large extent, this need can be addressed by the development of imaging techniques [22] as well as motion tracking using appropriate technical devices, including those based on 3D systems or wearable inertial sensors, which make it possible to record human motions.

The upper limb movement analysis is essential to objectively monitor rehabilitation interventions and contribute to improving the overall treatment outcomes. Motion quantification using kinematic analysis can help to understand the mechanisms underlying functional improvement following an intervention. For this purpose, the discriminant analysis of movements of post-stroke patients with healthy controls of similar age was used. The systematically determined and statistically selected features of the acquired upper limb motion data treated as time series were used. The data were obtained from the OptiTrack optometric system.

## 2. Materials and Methods

The assumption of this work is the achievement of objective results of the patient’s movement structure, which forms the basis for in-depth analysis and, consequently, for determining the upper limb movement that are most affected by stroke compared to healthy people [21,23]. For such a need, the analysis of relevant and systematically identified features of upper limb movement in post-stroke adults is presented. For this purpose, the basic features were calculated using movements defined by the x, y, and z coordinates (i.e., 3D trajectory) and compared to the results of post-stroke patients with healthy controls of similar age.

**Figure 1:**
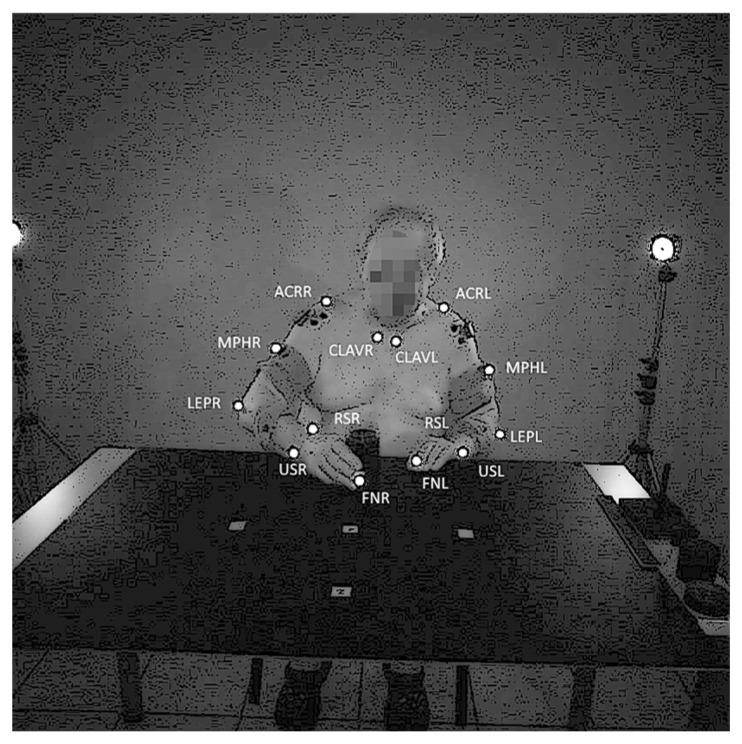
Markers scheme during the recording of the drinking task by the post-stroke participant. Markers lokations: CLAVR/L—clavicular heads, ACRR/L—acromion process, MPHR/L—middle part of the humeri, LEPR/L—lateral epicondyle, RSR/L—radial styloid, USR/L—ulnar styloid, FNR/L—index finger nails, R/L—right/left upper limb.

The upper limb kinematic chain monitored by the system included three classes as follows: the non-affected (G0) and the affected (G1) upper limb in stroke patients; the dominant and non-dominant upper limb in healthy controls (G2). The study involved a large set of time signal features to describe the statistical differences among the three data classes. The kinematic model of the upper limb consisted of seven markers on each side of the body (Figure 1) [24,25]. Completion of the grasping and drinking task was recommended for people with moderate to mild hemiparesis (32 out of 66 upper limb (FM-UE) scores using the Fugl-Meyer Assessment) [26,27]. A total of 54 participants were recruited for the study, including 35 stroke patients (stroke group) and 19 healthy individuals (control group). The inclusion and exclusion criteria were the same as those used in our earlier studies [24,25]. Among the 35 participants affected by ischemic stroke, there were 16 women and 19 men (mean age 67 ± 8.9 years). These patients were treated 3–16 months after their first stroke. Stroke type was as follows: PACI (parietal anterior cerebral ischemia)—23; TACI (total anterior cerebral ischemia)—10; LACI (lacunar cerebral ischemia)—2. Time after stroke (days) 287.8/40.8. The control group consisted of 19 recruited healthy participants, whose ages matched the post-stroke patients, and included 14 women and 5 men (mean age: 64 ± 9.0 years). The following criteria were met for the post-stroke group of participants: spasticity ≤ 2, in accordance with the modified Ashworth scale, who were able to stretch the affected arms out; no apraxia or shoulder pain that may interfere with task accomplishment; no neuromuscular, orthopedic disorders, major visual attention problems, or major perceptual or cognitive deficits; ability to provide informed consent; disturbances in cognitive functions measured by the Mini-Mental State Examination (MMSE ≥ 24); NIHSS (National Institutes of Health Stroke Scale) total score: median 4 (2–5) [24,25].

The experiment constituted an observational study of chronic stroke patients with mild to moderate upper limb motor impairment. All participants received detailed information regarding the experimental procedure and gave their written consent to participate, and informed consent was obtained from all subjects and/or their legal guardian(s). The research project was approved by the Bioethics Committee of the Opole Medical Chamber (No. 215, 25 March 2015), and the study was conducted in accordance with the Helsinki Declaration recommendations for clinical trials on humans.

### Study Protocol

The study was carried out at the Rehabilitation Department, Hospital Saint Roch in Ozimek, Poland. The study took place in a specially prepared room equipped with a table and a chair in the middle and eight OptiTrack high-speed and high-resolution cameras. The main advantage of the OptiTrack system is its mobility because it consists of cameras placed on a tripod, which can be set up in any place in accordance with the procedure described in the system documentation [28]. The markers were placed in accordance with the upper body marker placement schema of the OptiTrack described in the system manual [29]. Markers were located on both upper limbs (right R/L left). Marker CLAVR/L (clavicular heads) was located at the end of the corresponding clavicle bone just above where the sternum starts. Marker ACRR/L (acromion process) was placed on top of each shoulder for the protruding bone. The prominence is usually located at the end of the corresponding clavicle bone just above where the upper arm starts. Marker MPHR/L (middle part of the humeri) was placed in the groove between the triceps muscles where skin movements are relatively minimal. Marker LEPR/L (lateral epicondyle) was placed on the medial side of the elbow axis. Marker RSR/L (radial styloid) was placed on the medial side of the wrist axis. Marker USR/L (ulnar styloid) was placed on the lateral side of the wrist axis. Marker FNR/L (index finger nails) was placed on the nail of the phalanx of the distal index finger.

The placement spots for the items were marked on the table. Before taking a seat at the table, each participant had markers glued directly to the skin. Next, an examiner performed a demonstration, and the participant was calibrated and placed behind the table. The participant remained sitting with a straight back and the upper arms bent at right angles in the elbow, thumbs under the edge of the table and the other fingers (2 to 5) extended while resting on the edge of the table. The participant was positioned at a forearm distance from the table. Then, the examiner placed the object to be lifted from the table on a marked spot. Before the lifting test, the participant received verbal instructions and then performed one try before the actual examination. Each participant performed three recorded movements for each upper limb (i.e., 6 movements). The protocol was performed similarly to the Frenchay Arm Test (FAT) and consisted of the following tasks: drinking from a glass, lifting a small and a large cylinder, closing and unscrewing a jar, removing a clip, combing hair, and drawing lines. Only 3 selected activities were analyzed. Three lifting motions were assessed as follows: (1) lifting a large cylinder (34 mm diameter, 7 cm long, weighing 450 g); (2) lifting a small cylinder (12 mm diameter cylinder, 5 cm long, weighing 190 g); and (3) drinking from a glass. These activities were assessed due to their simplicity, repeatability, and ease of analysis [30].

Activity 1 and 2 consisted of the following steps: grasp the cylinder, set it on its side approximately 15 cm from the table edge, lift it as high as possible (preferably to extend the upper limb in the elbow joint), and replace it without dropping. Activity 3 was as follows: pick up a glass, positioned about 15 to 30 cm from the edge of the table; the first phase was to reach out for the glass from the starting position and then to grasp and bring the glass close to the mouth to drink, and to place it back on the table behind a marked line, followed by returning the glass to the initial position.

## 3. Results

### Data Analysis

Motion data were recorded using an optical motion capture system and used for further analysis in a time series of upper limb kinematic chain marker positions in a 3D space. A time series is a sequence of observations taken sequentially over time [27]. In order to use a set of time series as input for supervised or unsupervised machine learning algorithms, each time series needs to be mapped into a well-defined feature space. The Python tsfresh package (time series feature extraction based on scalable hypothesis tests) was used to systematically identify and extract meaningful features from the time series. The relevant features were selected on the basis of automatically configured hypothesis tests (FRESH algorithm) with respect to the multiclass classification problem with defined classes G0, G1, and G2 [31]. Automated feature extraction using a vast number of possibly meaningful statistics can be applied in the context of biomechanical data analysis [32]. However, the extracted features are often nested, complex, and hard to interpret, and thus show limited comparability. Our proposal is a quantitative analysis of the selected significant systematically extracted features.

**Figure 2:**
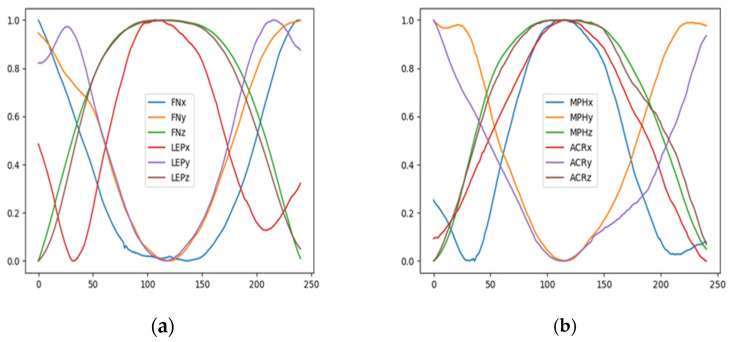
Cropped and normalized marker trajectory signals during the lifting of a large cylinder of one participant from class G0 (non-affected participant’s upper limb). Left plot (**a**)—trajectory of FN and LEP markers. Right plot (**b**)—trajectory of MPH and ACR markers.

The data acquired by OptiTrack were the position data (following values of the x, y, and z coordinates) of the FN, LEP, MPH, and ACR markers constituting the kinematic chain of the upper limb. Captured motion signals were recorded at a frequency of 100 Hz. The time series was cropped to raise an upper limb only. The z-axis (longitudinal axis) represents the up and down motions. Based on the change in the z-coordinate value of the FN marker, only the lifting and lowering of upper limb data were cropped. Figure 2a,b show exemplary time series during the lifting of a large cylinder. From all input time series, only samples between the start and end times of the activity based on the change in FNz values (z coordinates of FN marker) were cropped. These are samples that had z coordinate value greater than the z value in the activity starting position plus the threshold value. The threshold value was 10% of the value of the entire range of FN marker movements along the z-axis during a given recording.

On the basis of such trajectories of the markers in 3D, the displacement, velocity, acceleration signals, displacement module, velocity module, and acceleration module signals were calculated. The module was calculated as the length of the vector. For example, the calculation for the magnitude of an FN marker acceleration is as follows:

$$FNA=\sqrt{{FNax}^{2}+{FNay}^{2}+{FNaz}^{2}}$$

The next step was normalization using the min-max method to a range of [0, 1]. The side of the movement was also unified through the sagittal plane (yz) reflection transformation of the right upper limb movement.

The input of the method used to systematically determine the features was a set of normalized time series (Table 1) for each recording of the upper limb that was lifting the object (small cylinder, large cylinder, and cup).

The input time series $T={\left\{\chi_{i}\right\}}_{1}^{60}$ was mapped into a feature space with a dimensionality of 794 and a feature vector $x_{i}=\left(x_{i,1},x_{i,2}\ldots ,x_{i,794}\right)$. Finally, 47,640 features were extracted. Such a set was prepared for each recording. An overview of the extracted features can be found in the tfresh documentation (https://tsfresh.readthedocs.io/en/latest/text/list_of_features.html (accessed on 5 February 2022). The prepared input data were divided into three data classes as presented in Table 2.

In the second step, each feature was individually and independently evaluated with respect to its significance in the classification task. The result of these tests was a vector of *p*-values, quantifying the significance of each feature for predicting the label (G0, G1, and G2) using nonparametric automated hypothesis tests. For the calculation of the feature significance for a real-valued feature, the Mann–Whitney U test was used, and for the binary-valued feature, the two-sided univariate Fisher test was used. For the classification of three defined labels, the selection problem was divided into three separate binary one-versus-rest classification problems. Next, the vector of *p*-values was evaluated on the basis of the multiple testing Benjamini–Yekutieli procedure with an FDR (false discovery rate) level equal to 0.05 [33] in order to decide which features to keep.

A total of 47,640 extracted features and 11,882 statistically significant features were found for discrimination class G2 from classes G0 and G1; 2022—for discrimination class G1 from G2 and G0; and 15,658—for discrimination class G0 from G2 and G1. The number of common features was 1004. Class G0 (non-affected upper limb) had the most different features from G1 (affected upper limb) and G2 (dominant and non-dominant upper limb in healthy controls) [25].

The detailed statistics of the significant common features are presented as follows: 338 features on the ACR marker, 459 features on the MPH marker, 104 features on the LEP marker, and 103 features on the FN marker. The movements of the ACR and MPH segments turned out to be the most differentiated.

With regard to the plane of movement, 368 features were recorded on the x-axis movement (transverse movements—left/right), 123 features on the y-axis movement (sagittal movements—forward/backward), and 241 features on the z-axis movement (vertical movements—up/down).

The most significant features for the movement on the x-axis were based on the ACR and MPH markers, and on the z-axis based on the FN marker.

With regard to the type of signals, 335 features were identified for the acceleration time series, 287 for velocity, 198 for displacement, and 184 for the trajectory time series. The most statistically significant features were determined on the basis of acceleration signals. The exact statistics of the significant features are presented in Table 3.

**Figure 3:**
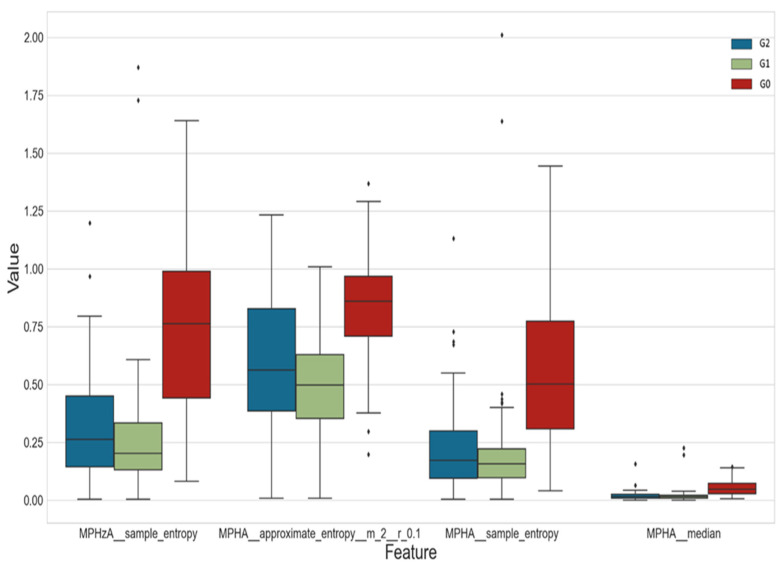
Boxplot of values of four exemplary features for classes G0, G1 and G2. Feature names are in accordance with the tsfresh systematic feature extraction algorithm and are listed in package documentation (https://tsfresh.readthedocs.io/en/latest/text/list_of_features.html (accessed on 5 February 2022).

Figure 3 presents a boxplot of four exemplary features that were significantly differentiated in the three defined classes.

## 4. Discussion

In the past, motion capture systems were adapted for rehabilitation procedures in the treatment of various diseases. Advances in machine learning and the emergence of newer motion capture solutions have contributed to the development of more automatic assessments of patient performance and recovery progress [25,33]. In the present study, the quantitative characteristics of the participants’ movements were assessed to generate the required trajectories and kinematics of the upper limb to accurately perform a functional task (lifting an object or lifting a cup to drink). Although these were simple activities, the required movement turned out to be dependent on the participant’s ability levels of body functions. The task of picking up items and drinking was chosen because it had already been learned, and could be easily standardized and accomplished with minimal investment in tracking equipment [21].

There is currently no consensus on the use of kinetic and kinematic measures (metrics) to restore mobility. In their analysis of 225 studies, Schwarz et al. found 151 different metrics used to measure upper limb movement [13].

The present study compared the performance of a simple ADL task by post-stroke patients (G0 and G1) with healthy people of similar age (G2). The numbers of identified statistically significant features were: 11,882 for the G2 class, 2022 for the G1 class, and 15,658 for the G0 class. The number of common features was 1004. The obtained results indicate that the upper arm movement (ACR and MPH markers) turned out to be the most differentiated. The most significant features were determined on the basis of the acceleration signals of the markers. The presented comparative analysis showed significant differences in the movement pattern, not only in relation to the limb affected by a stroke but also showed that the upper limb not affected by a stroke is motorically different from the upper limb of a healthy person of the same age. This indicates a motor disorder in both upper limbs in stroke patients. In the present study, the measurement of acceleration turned out to be an important tool for assessing differences in the activities studied. In practice, such a solution can be obtained by using a simple accelerometer attached to the upper arm. According to the present research, this segment of the body is most sensitive to changes in the trajectory of movement, especially in abduction and adduction movements.

The Computational Approaches to Patient Performance Assessment in Rehabilitation Programs using Motion Capture Systems may play a key role in complementing traditional rehabilitation assessments by trained clinicians and in assisting patients in home-based rehabilitation. The computational methods for assessing exercises discussed in the literature are usually grouped into three main categories: discrete motion score, rule-based, and template-based approaches [33]. The ideal solution would incorporate systems based on the location of one sensor, or a maximum of three sensors, on the patient’s body, due to which trained staff would be able to track the patient’s progress. Thus, the determination of the most differentiated segment of the body may be a sensitive measure of changes taking place following the applied rehabilitation methods.

Upper limb movement restoration during stroke recovery is a particularly important pillar of rehabilitation practice. An increasing number of popular methods are based on wearable sensors, especially those based on accelerometers, and the research shows that the accelerometer signal can be best used on the MPH segment than on popular wrists. In fact, rehabilitating upper limb movements is usually much more difficult compared to lower limb movements. For these reasons, researchers have been developing new methods and technologies to make the assessment modalities more accurate, faster, and easier for the patient to accept [34,35,36,37]. Thus, any motor control strategy must be capable of guiding the entire kinematic chain in such a way as to achieve the desired movement and compensate for any movement disturbances, e.g., the onset of muscle fatigue, as well as a strong non-linear and time-varying musculoskeletal response [37,38,39]. In addition, with the current development of motion-tracking techniques, we have better tools to assess the functional status of patients. This creates the possibility of an objective and accurate qualitative and quantitative assessment of the human movement. An ideal solution would be to create simple solutions based on MoCap systems that can be used in everyday therapeutic practice.

The present research shows that the brachial area is the most sensitive site to changes in the movement pattern, and the abduction and adduction movements proved to be most sensitive to movement changes. Brain repair is best represented by fine-grained quantification of motion, which is sensitive and specific, i.e., capable of picking up small but real changes in behavior. To date, there has been disagreement on how to use kinematics and kinetics to achieve this goal. Researchers suggest that only by measuring these movement traits can neural changes associated with behavior restitution be distinguished from compensatory strategies [21,39].

## 5. Conclusions

In the present study, the 1004 identified features distinguished three statistically significant classes of the upper limb (G0, G1, and G2). Analyzing the features in terms of segment type, most were concerned with the arm (MPH marker). Taking into account the type of data, the most important features were determined on the basis of acceleration signals. As for the movement plane, the most significant features were movements in the frontal plane (arm abduction and adduction). The present study indicates that arm abduction and adduction movements require special attention from therapists, in both the affected and non-affected limbs. In addition, the obtained differences in acceleration indicate the possibility of monitoring the progress of rehabilitation through the use of an acceleration sensor placed on the arm.

## Figures and Tables

**Table 1:** Set of determined time series (T).

Marker Signals	FNR, FNL	LEPR, LEPL	MPHR, MPHL	ACRR, ACRL
3D trajectories (coordinates) of markers	FNx, FNy, FNz	LEPx, LEPy, LEPz	MPHx, MPHy, MPHz	ACRx, ACRy, ACRz
3D displacement of markers	FNdx, FNdy, FNdz	LEPdx, LEPdy, LEPdz	MPHdx, MPHdy, MPHdz	ACRdx, ACRdy, ACRdz
Displacement module	FND	LEPD	MPHD	ACRD
3D velocities of markers	FNvx, FNvy, FNvz	LEPvx, LEPvy, LEPvz	MPHvx, MPHvy, MPHvz	ACRvx, ACRvy, ACRvz
Speed	FNV	LEPV	MPHV	ACRV
3D accelerations of markers	FNax, FNay, FNaz	LEPax, LEPay, LEPaz	MPHax, MPHay, MPHaz	ACRax, ACRay, ACRaz
Acceleration module	FNA	LEPA	MPHA	ACRA

**Table 2:** The division of recordings into classes.

Class	Number of Participants	Recorded Upper Limb	Number of Recordings
G0	35 after stroke	non-affected	105
G1	35 after stroke	affected	105
G2	19 control group	the left and right healthy upper limb	114

**Table 3:** The number of significant features depending on the maker, axis of movement, and type of time series.

	Module		Trajectory		Acceleration		Velocity		Displacement	
**ACR**	**38**	**A**	**52**	**X**	26	X	29	X	42	X
	35	V	0	Y	14	Y	9	Y	11	Y
	13	D	0	Z	35	Z	30	Z	4	Z
**MPH**	62	A	47	X	34	X	42	X	37	X
	62	V	6	Y	12	Y	7	Y	30	Y
	15	D	14	Z	35	Z	53	Z	3	Z
**LEP**	18	A	31	X	2	X	3	X	14	X
	7	V	3	Y	3	Y	0	Y	3	Y
	3	D	11	Z	4	Z	0	Z	2	Z
**FN**	7	A	1	X	5	X	3	X	0	X
	0	V	8	Y	11	Y	0	Y	6	Y
	12	D	11	Z	29	Z	7	Z	3	Z

(ACR—acromion process, MPH—middle part of the humeri, LEP—lateral epicondyle, FN—index finger nails, A—acceleration; V—velocity; D—displacement. Module—length of 3D vector).

## Data Availability

The data that support the findings of this study are available from the first author and the corresponding authors upon reasonable request.
